# Supplementary material for: Association of single nucleotide polymorphisms in ITLN1 gene with ischemic stroke risk in Xi’an population, Shaanxi province
Source: PeerJ. 2024 Mar 22;12:e16934. doi: 10.7717/peerj.16934 (PMC10962333; doi:10.7717/peerj.16934)
Supplement: Supplemental Information 1 [file peerj-12-16934-s001.docx]

Online Resource 1 The Primer sequence of DNA fragment in the promoter region of *ITLN1*

| Primers | Base sequence |
| --- | --- |
| Primer F | 5’-TGTGTAGAGGTAAACGACCAAGAATAAC-3’ |
| Primer R | 5’-CATTGTAATCTAAAGAAAGCAAGATGAAGG-3’ |

Online Resource 2 In silico analysis for the selected SNPs function annotation

| SNP-ID | RegulomeDB Score | HaploReg |
| --- | --- | --- |
|  |  |  |
| rs77824633 | [4](http://legacy.regulomedb.org/snp/chr1/160854748) | DNAse, Proteins bound, Motifs changed |
| rs79209815 | [3a](http://legacy.regulomedb.org/snp/chr1/160854926) | DNAse, Motifs changed |
| rs112656766 | [3a](http://legacy.regulomedb.org/snp/chr1/160854935) | DNAse, Motifs changed |
| rs12094513 | [6](http://legacy.regulomedb.org/snp/chr1/160855413) | Motifs changed |
| rs12094649 | [6](http://legacy.regulomedb.org/snp/chr1/160855413) | Motifs changed |
| rs6427553 | No Data | Selected eQTL hits |
| rs12094703 | No Data | Motifs changed |
| rs12091185 | [6](http://legacy.regulomedb.org/snp/chr1/160855413) | Motifs changed |
| rs7411035 | No Data | Motifs changed, Selected eQTL hits |
| rs74547401 | [6](http://legacy.regulomedb.org/snp/chr1/160855413) | Motifs changed |
| rs4656958 | [5](http://legacy.regulomedb.org/snp/chr1/160856963) | DNAse, Motifs changed, Selected eQTL hits |
| rs58170012 | No Data | Motifs changed |
| rs111321091 | No Data | - |
| rs80262455 | [2b](http://legacy.regulomedb.org/snp/chr1/160857669) | Proteins bound, Motifs changed |

2b indicates that the variant is likely to affect binding.

3a indicates that the variant is less likely to affect binding.

4, 5 and 6 indicate that the variant has minimal binding evidence
